# Pembrolizumab and Chemotherapy Combination Prolonged Progression-Free Survival in Patients with NSCLC with High PD-L1 Expression and Low Neutrophil-to-Lymphocyte Ratio

**DOI:** 10.3390/ph15111407

**Published:** 2022-11-14

**Authors:** Jeng-Shiuan Tsai, Sheng-Huan Wei, Chian-Wei Chen, Szu-Chun Yang, Yau-Lin Tseng, Po-Lan Su, Chien-Chung Lin, Wu-Chou Su

**Affiliations:** 1 Department of Internal Medicine, National Cheng Kung University Hospital, College of Medicine, National Cheng Kung University, Tainan 704, Taiwan; 2 Department of Surgery, National Cheng Kung University Hospital, College of Medicine, National Cheng Kung University, Tainan 704, Taiwan; 3 Institute of Clinical Medicine, National Cheng Kung University Hospital, College of Medicine, National Cheng Kung University, Tainan 704, Taiwan; 4 Department of Biochemistry and Molecular Biology, College of Medicine, National Cheng Kung University, Tainan 704, Taiwan; 5 Center of Applied Nanomedicine, National Cheng Kung University, Tainan 704, Taiwan; 6 Department of Oncology, National Cheng Kung University Hospital, College of Medicine, National Cheng Kung University, Tainan 704, Taiwan

**Keywords:** non-small cell lung cancer, pembrolizumab, neutrophil-to-lymphocyte ratio

## Abstract

The use of immune checkpoint inhibitors (ICIs) has provided overall survival (OS) benefits in patients with treatment-naïve advanced non-small cell lung cancer (NSCLC) without targetable driver mutations. However, studies comparing ICIs monotherapy with combination therapy either with chemotherapy or radiotherapy in programmed death-ligand 1 high expressors remain limited. This study aimed to retrospectively compare the treatment efficacy of the therapies by studying 47 patients with treatment-naïve advanced NSCLC who received ICI monotherapy (*n* = 28) or combination therapy either with chemotherapy or radiotherapy (*n* = 19). Progression-free survival (PFS) and OS were estimated using the Kaplan–Meier method and compared using log–rank tests. It was observed that patients who received combination therapy had a better PFS than monotherapy, but no such significant benefit was observed in OS. The difference in PFS was higher in the subgroup of patients with low neutrophil-to-lymphocyte ratio (NLR) than in the high-NLR patient subgroup. This study suggests that pembrolizumab in combination with chemotherapy or radiotherapy could provide a significant benefit in PFS, especially in patients with treatment-naïve advanced NSCLC with low NLR. Furthermore, our study also demonstrates the potential use of NLR as a biomarker for prediction of treatment outcomes in patients with advanced NSCLC receiving combination therapy.

## 1. Introduction

Non-small cell lung cancer (NSCLC) accounts for approximately 80–85% of all lung cancers and is the leading cause of cancer-related death worldwide [1]. Although targeted therapy has provided a better treatment response, nearly half of the patients do not have targetable driver mutations [2]. In the past five years, there has been growing evidence regarding the role of immune checkpoint inhibitors (ICIs) in patients with advanced-stage NSCLC [3]. The phase III KEYNOTE-024 study demonstrated that pembrolizumab monotherapy could provide an overall survival (OS) benefit in patients with advanced stage NSCLC, who exhibit a high expression of programmed death-ligand 1 (PD-L1) [4]. This result was further confirmed by subgroup analysis of another phase III study, KEYNOTE-042 [5]. Similarly, the Impower 110 study trial also revealed that atezolizumab provided OS benefits in patients with advanced stage NSCLC and high PD-L1 expression [6]. However, the objective response rate (ORR) in ICIs monotherapy was observed to be low, suggesting that there is still room for improvement.

Combination therapy, either with radiotherapy or chemotherapy, has been well investigated for improving therapeutic outcomes. Theoretically, both radiotherapy and chemotherapy can increase neoantigen presentation and induce subsequent immunogenic cell death [7]. In a pooled analysis of two phase II clinical trials, the addition of radiotherapy to ICIs monotherapy significantly improved progression-free survival (PFS), OS, and ORR [8]. Moreover, the KEYNOTE-189 and KEYNOTE-407 studies also demonstrated that the first-line combination of pembrolizumab and chemotherapy provided longer OS benefits in advanced-stage NSCLC regardless of PD-L1 expression level [9,10]. Furthermore, it was observed that patients who received chemoimmunotherapy (a combination of chemotherapy and pembrolizumab, an immunotherapeutic drug) showed significant improvement in PFS in the first three months [9], compared with patients who received pembrolizumab monotherapy [4,5]. Although the OS outcome was observed to be better in patients who received chemoimmunotherapy, both pembrolizumab monotherapy and combination therapy with pembrolizumab and chemotherapy is recommended for patients with high PD-L1 expression. Therefore, this points to a need for a prospective randomized controlled trial comparing the treatment efficacy of pembrolizumab alone or in combination with chemotherapy.

Recently, cohort studies comparing treatment efficacy of pembrolizumab alone or in combination with chemotherapy have been carried out, but no significant difference in survival outcomes was observed amongst patients who received different treatment modalities [11,12,13,14]. The possible reasons for the insignificant difference might be attributed to the higher toxicities in combination therapy [14] and its limited clinical benefits in certain patient subgroups [11]. Thus, to identify the optimal subgroup of patients who are more susceptible to immunotherapy, an increasing number of studies have investigated the role of the neutrophil-to-lymphocyte ratio (NLR) in patients who received immunotherapy [15,16]. We hypothesized that NLR could also serve as a predictive biomarker in patients who received chemoimmunotherapy. Therefore, in the present study, we aimed to compare the efficacy of pembrolizumab monotherapy and combination therapy and identify a subgroup of patients who might have benefited from this combination therapy.

## 2. Results

### 2.1. Patient Characteristics

**Figure 1:**
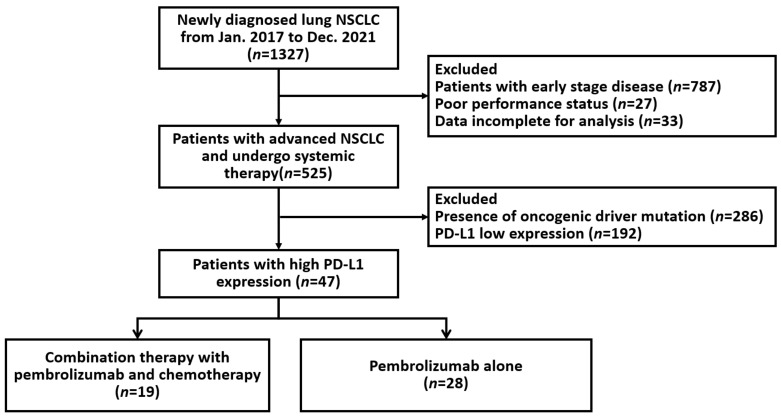
Flow chart describing enrollment of patients in the study. NSCLC, non-small cell lung cancer; PD-L1, programmed death ligand-1.

A total of 47 patients who received first-line pembrolizumab were enrolled in the study, which included 28 who received monotherapy and 19 who received combination therapy. Figure 1 illustrates the flowchart of patient enrollment. All patients were included in PFS and OS analyses. The baseline characteristics of all the patients, which were similar between patients in monotherapy group and combination group, are summarized in Table 1. All patients had metastatic disease, the median age of the patients was 71 years (interquartile range: 63–77 years) and included 35 males (74.5%) and 12 females (25.5%). The histological types were non-squamous in 36 (76.6%) patients and squamous in 11 (23.4%) patients. Twelve patients (25.5%) had brain metastasis. Regarding combination therapy, 11 patients (23.4%) received chemotherapy, and 8 patients (17%) received radiotherapy. The detailed information of combined radiotherapy was summarized in Supplementary Table S1.

### 2.2. Progression-Free Survival and Overall Survival

**Figure 2:**
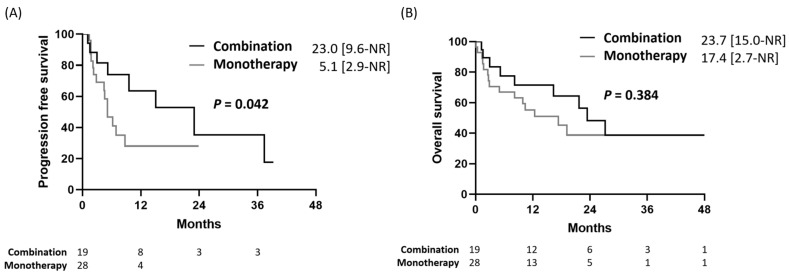
The (**A**) progression-free survival and (**B**) overall survival in patients who received combination therapy or pembrolizumab monotherapy. NR—not reached.

The median follow-up duration was 7.2 months. Patients who received combination therapy had a median PFS of 23.0 months (interquartile range [IQR] 9.6–not achieved [NA]), which was significantly longer than that of patients who received pembrolizumab monotherapy (5.1 months, IQR 2.9–NA) (*p* = 0.042, Figure 2A). Using Cox proportional hazards regression to adjust for possible confounders, combination therapy with pembrolizumab and chemotherapy was an independent good prognostic factor for PFS (hazard ratio [HR] 0.17, 95% CI: 0.05–0.26, *p* = 0.006) (Table 2). Patients who received combined pembrolizumab and radiotherapy also had marginally better PFS (HR 0.26, 95% CI: 0.05–1.30), than those who received pembrolizumab monotherapy, but the difference was not statistically significant (*p* = 0.101); this could be attributed to the limited patient number. In addition, patients who were current smokers also had better PFS (hazard ratio [HR] 0.07, 95% CI: 0.01–0.48, *p* = 0.007) (Table 2) than non-smokers.

In contrast, even though patients who received combination therapy had an OS of 23.7 months (IQR 15.0–NA), which was numerically longer than patients who received pembrolizumab monotherapy (17.4 months, IQR 2.7–NA), no statistical significance was observed (*p* = 0.384, Figure 2B). Using Cox proportional hazards regression to adjust for possible confounders, combination therapy still had no impact on OS, regardless of chemotherapy or radiotherapy (Table 3).

### 2.3. Subgroup Analysis

**Figure 3:**
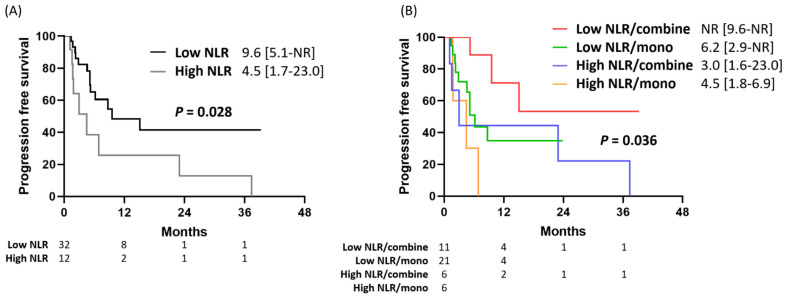
(**A**) The progression-free survival among patients with high/low NLR. (**B**) The progression-free survival among patients with high/low NLR and received combination/monotherapy. NA— not achieved.

In the subgroup analysis, patients were classified into a high NLR group (NLR ≥ 8) and a low NLR group (NLR < 8). Patients with a low NLR had a significantly longer PFS than those with a high NLR (9.6 vs. 4.5 months, *p* = 0.028) (Figure 3A). In the subgroup of patients with a low NLR, the PFS of patients who received combination therapy was not achieved (IQR 9.6–not achieved), was significantly longer than that of patients who received pembrolizumab monotherapy (6.2 months, IQR 2.9–not achieved) (*p* = 0.036, Figure 3B). In contrast, in the subgroup of patients with a high NLR, the PFS was similar between patients who received combination therapy and those who received immunotherapy monotherapy (Figure 3B).

## 3. Discussion

The present study showed that the first-line pembrolizumab and chemotherapy combination had superior PFS than pembrolizumab monotherapy in patients with advanced NSCLC and high PD-L1 expression (>50%). However, the clinical benefit of PFS did not translate into OS benefit. In subgroup analysis, the PFS benefit from combination therapy was more significant in patients with a low NLR.

Comparison of treatment efficacy between combination therapy and ICIs monotherapy has been widely studied. In a meta-analysis of four phase III clinical trials, combination therapy demonstrated longer PFS and similar OS compared with pembrolizumab monotherapy. Although the study results favored combination therapy, subsequent real-world studies demonstrated similar PFS and OS among patients who received different treatment modalities [11,12,14]. On the contrary, a multicenter retrospective cohort study conducted by Matsumoto et al. showed that pembrolizumab monotherapy provided longer PFS than combination therapy in the subgroup of patients with metastases to the liver, lung, adrenal glands, bone, or lymph nodes [13]. Moreover, a retrospective study using electronic databases of four Israeli cancer centers showed that pembrolizumab monotherapy and combination therapy with pembrolizumab and chemotherapy provided similar OS in patients with high PD-L1 expression after propensity score matching, and a significantly longer median OS was observed only in female patients who received the combination therapy [11]. Therefore, these studies highlight that combination therapy might provide survival benefits in certain subgroups of patients, which is similar to the results obtained in the present study, where we demonstrated a PFS benefit in patients who received combination therapy with pembrolizumab and chemotherapy, especially in patients with a low NLR. Therefore, the results of this study could help in guiding the selection of treatment strategies for patients with advanced-stage NSCLC with high PD-L1 expression.

Both chronic inflammation and adaptive immune surveillance are well-established cancer hallmarks [17]. NLR in the peripheral blood could be recognized as a surrogate marker for both inflammation status (high NLR) and adaptive immune surveillance (low NLR). In a retrospective cohort study that enrolled 1,714 patients across 16 different cancer types, it was observed that patients with higher NLR had significantly shorter PFS and OS following treatment with ICIs [18]. The prognostic role of the NLR in patients with NSCLC receiving immunotherapy has also been well investigated. In a retrospective study of 133 patients with PD-L1-unselected stage IIIB-IV NSCLC treated with PD-1 inhibitors, NLR was shown to be an independent prognostic factor for PFS [16]. Furthermore, studies focusing on patients with NSCLC with high PD-L1 expression who received pembrolizumab monotherapy also demonstrated that a low NLR was associated with favorable outcomes [15,19]. Additionally, with multiplexed immunofluorescence to investigate immunophenotype, patients who exhibited a derived neutrophil-to-lymphocyte ratio (dNLR) of <2.6 had significantly greater infiltration of tumor-associated CD8+, FOXP3+, PD-1+ immune cells, and PD-1+ CD8+ T cells in tumor tissue than those with a dNLR ≥ 2.6 [20]. Although the NLR-based blood biomarker score could help in predicting the treatment outcome in patients who received combination therapy with pembrolizumab and chemotherapy [21], no studies have been carried out focusing on its use in the treatment selection between combination therapy and pembrolizumab monotherapy. In the present study, we demonstrated that a low NLR could predict a better treatment response to combination therapy in patients with NSCLC with high PD-L1 expression.

The addition of radiotherapy to immunotherapy could enhance the occurrence of abscopal responses and hence, improve outcomes [22]. Several preclinical studies have reported that tumor irradiation increases tumor antigen release, diversifies the T-cell receptor (TCR) repertoire of tumor-infiltrating T cells, increases activation of cytotoxic T cells, and reduces tumor-infiltrating myeloid-derived suppressor cells [23,24,25,26]. In a multicenter randomized phase II study (PEMBRO-RT) which enrolled 92 patients with NSCLC, the combination of radiotherapy was shown to be able to increase the ORR to 50% despite only a marginal benefit in survival outcome due to the limited patient number [27]. Similarly, in another phase I/II study conducted by the MD Anderson Cancer Center (MDACC), the combination of radiotherapy also demonstrated numerical improvement in PFS [28]. In the pooled analysis of the PEMBRO-RT [27] and MDACC trials [28], to overcome the limitation of patient number, it was observed that the addition of radiotherapy could significantly improve the PFS, OS, and ORR [8]. In our clinical data analysis, although the hazard ratio (HR) of adding radiotherapy was 0.26, the clinical benefit was not significant owing to the limited number of patients. A larger prospective study is needed to validate these results.

There are some limitations to the present study. First, it was a retrospective study conducted in a single tertiary referral center, and the limited number of patients precluded definitive conclusions. Therefore, a prospective study is warranted to investigate the survival benefits of chemoimmunotherapy. Second, the baseline characteristics were unbalanced between patients who received pembrolizumab monotherapy and those who received combination therapy, which may have interfered with the study results. However, we used COX proportional hazard regression analysis to adjust for all possible confounding factors, and the use of combination therapy was still an independent prognostic factor for PFS. Third, the underlying genomic alterations, including P53, KRAS, STK11 and KEAP1, which might affect the efficacy of immunotherapy were not assessed [29] even though studies regarding the role of co-occurring mutations in chemotherapy–immunotherapy combinations remain limited [30]. Additionally, these genomic alterations are more likely to be prognostic factors than predictive biomarkers [31], and the interaction between genomic alteration and the tumor–immune microenvironment requires further investigation. In the present study, we used NLR as a biomarker, which could reflect the balance between inflammatory response and adaptive immunity, and could be a surrogate for the tumor–immune microenvironment with potentially better predictability.

## 4. Materials and Methods

### 4.1. Patients

Patients with treatment-naïve advanced NSCLC who received pembrolizumab as first-line therapy at a tertiary referral center from January 2017 to August 2021 were retrospectively enrolled in the study. Patients with low or no PD-L1 expression (PD-L1 < 50%) and patients who had targetable driver mutations were excluded from the study. All patients underwent computed tomography (CT) of the chest, a magnetic resonance imaging (MRI) of the brain, and a whole-body bone scan for complete staging, based on the tumor, node, metastasis (TNM) system proposed by the American Joint Committee on Cancer, 8th edition. The immune checkpoint inhibitor pembrolizumab was administered as monotherapy or combination therapy at the physicians’ discretion. The baseline characteristics of these patients were recorded, including age, sex, performance status, histological subtype, smoking status, presence of brain metastasis and TNM stage. All data were anonymized according to approved guidelines and the Declaration of Helsinki. This study was approved by the institutional ethics committee of National Cheng Kung University Hospital (IRB number: B-ER-109-344).

### 4.2. PFS and OS Analysis

After treatment initiation, all patients underwent chest computed tomography every 12 weeks to evaluate tumor response. PFS was calculated from the date of treatment initiation until the date of radiological progression discontinuation due to adverse events, or death, according to the Response Evaluation Criteria in Solid Tumors v1.1 [32]. Censoring was applied on the date of the last follow-up in the absence of disease progression. OS was calculated from the initiation of treatment until death. In the subgroup survival analysis, patients were classified based on pretreatment neutrophil-to-lymphocyte ratio (NLR), and PFS was compared between patients who received combination therapy and those who received immunotherapy alone.

### 4.3. Statistical Analysis

The frequencies and descriptive statistics of the demographic and clinical variables were calculated. Categorical variables were compared using the chi-square test or Fisher’s exact test, whereas continuous variables were compared using the student’s t-test or the Wilcoxon rank–sum test. The PFS and OS of all patients were estimated using the Kaplan–Meier method and compared using the log–rank test. Cox proportional hazards regression analysis was performed to identify the predictors of PFS and OS. The selection of predictors and determinants was based on previous studies that investigated the prognostic factors of survival [33]. Statistical analyses were performed using the SAS version 9.4 software (SAS Institute, Cary, NC, USA). All reported *p* values were two-sided and a *p*-value of lower than 0.05 is considered as statistically significant.

## 5. Conclusions

In this study, we showed that among patients with advanced-stage NSCLC and high PD-L1 expression, combination therapy with pembrolizumab and chemotherapy provided significantly better PFS, but not OS, than pembrolizumab monotherapy. Moreover, the survival benefit was more prominent in patients with low NLR, suggesting the use of NLR as a potential biomarker for prediction of treatment outcomes. However, further prospective studies are required to validate these results.

## Figures and Tables

**Table 1:** Clinical characteristics of all patients.

Characteristic	Total Population (%)	Pembrolizumab Monotherapy	Combination Therapy	*p* Value
	*n* = 47	*n* = 28	*n* = 19	
Age	71 (63–77)	73 (66–80)	69 (62–77)	0.246
<65 y/o	13 (27.6%)	6	7	
≥65 y/o	34 (72.4%)	22	12	
Gender				0.919
Female	12 (25.5%)	7	5	
Male	35 (74.5%)	21	14	
Smoking				0.188
Smoker	13 (27.6%)	9	4	
Non-smoker	21 (44.8%)	14	7	
Ex-smoker	13 (27.6%)	5	8	
ECOG PS				0.705
0–1	41 (87.2%)	24	17	
>2	6 (12.8%)	4	2	
Histology				0.698
Non-squamous NSCLC	36 (76.6%)	22	14	
Squamous cell carcinoma	11 (23.4%)	6	5	
Brain metastasis				0.143
Yes	12 (25.5%)	5	7	
No	35 (74.5%)	23	12	
Treatment modality				
Pembrolizumab	28 (59.5%)	28	0	
Pembrolizumab plus chemotherapy	11 (23.4%)	0	11	
Pembrolizumab plus radiotherapy	8 (17.0%)	0	8	

ECOG, Eastern Cooperative Oncology Group; NSCLC, non-small cell lung cancer; PS, performance status.

**Table 2:** Cox proportional hazard regression analysis of PFS.

	Hazard Ratio 95% CI	*p*
Age		
≥65 vs. <65 y/o	0.63 (0.20–2.01)	0.437
Gender		
Male vs. female	1.78 (0.52–6.14)	0.36
Smoking		
Smoker vs. non-smoker	0.07 (0.01–0.48)	0.007
Ex-smoker vs. non-smoker	1.38 (0.40–4.73)	0.608
ECOG PS		
≤1 vs. >2	1.83 (0.44–7.51)	0.402
Histology		
Non-squamous vs. squamous NSCLC	0.47 (0.13–1.74)	0.26
Brain metastasis		
Presence vs. Absence	0.98 (0.23–3.21)	0.97
Treatment		
Pembrolizumab plus chemotherapy vs. pembrolizumab monotherapy	0.17 (0.05–0.62)	0.006
Pembrolizumab plus radiotherapy vs. pembrolizumab monotherapy	0.26 (0.05–1.30)	0.101

ECOG, Eastern Cooperative Oncology Group; NSCLC, non-small cell lung cancer; PS, performance status; PFS, progression-free survival.

**Table 3:** Cox proportional hazard regression analysis of OS.

	Hazard Ratio 95% CI	*p*
Age		
≥65 vs. <65 y/o	1.79 (0.61–5.26)	0.293
Gender		
Male vs. female	1.51 (0.43–5.35)	0.523
Smoking		
Smoker vs. non-smoker	0.73 (0.21–2.46)	0.605
Ex-smoker vs. non-smoker	1.081 (0.31–3.81)	0.904
ECOG PS		
≤1 vs. >2	2.02 (0.51–8.07)	0.318
Histology		
Non-squamous vs. squamous NSCLC	1.20 (0.45–3.18)	0.711
Brain metastasis		
Presence vs. absence	0.93 (0.33–2.60)	0.892
Treatment		
Pembrolizumab plus chemotherapy vs. pembrolizumab monotherapy	0.66 (0.22–1.97)	0.457
Pembrolizumab plus radiotherapy vs. pembrolizumab monotherapy	0.81 (0.24–2.78)	0.742

ECOG, Eastern Cooperative Oncology Group; NSCLC, non-small cell lung cancer; PS, performance status; OS, overall survival.

## Data Availability

Data is contained within the article and Supplementary Material.

## Supplementary Materials

The following supporting information can be downloaded at: https://www.mdpi.com/article/10.3390/ph15111407/s1, Table S1: The detailed information of combined radiotherapy.
