# Supplementary material for: Pembrolizumab and Chemotherapy Combination Prolonged Progression-Free Survival in Patients with NSCLC with High PD-L1 Expression and Low Neutrophil-to-Lymphocyte Ratio
Source: Pharmaceuticals (Basel). 2022 Nov 14;15(11):1407. doi: 10.3390/ph15111407 (PMC9694359; doi:10.3390/ph15111407)
Supplement: Supplementary file 1 [file pharmaceuticals-15-01407-s001.zip › pharmaceuticals-1988543-supplementary.pdf]

Supplementary Table S1. The detailed information of combined radiotherapy

**Case number**	**Radiotherapy**	**Location and Dosage**
**1**	Palliative	Whole brain (3000 cGy)
**2**	Palliative	Primary tumor (3000 cGy)
**3**	Palliative	Whole brain (3000 cGy), Bone metastasis (3000 cGy)
**4**	Palliative	Primary tumor (2000 cGy)
**5**	Palliative	Primary tumor (3000 cGy)
**6**	Palliative	Primary tumor (3000 cGy), Whole brain (3000 cGy)
**7**	Palliative	Primary tumor (3000 cGy)
**8**	Palliative	Primary tumor (3000 cGy)
